# Carrier multiplication detected through transient photocurrent in device-grade films of lead selenide quantum dots

**DOI:** 10.1038/ncomms9185

**Published:** 2015-09-08

**Authors:** Jianbo Gao, Andrew F. Fidler, Victor I. Klimov

**Affiliations:** 1 Center for Advanced Solar Photophysics, Chemistry Division, Los Alamos National Laboratory, Los Alamos, New Mexico 87545, USA.

## Abstract

In carrier multiplication, the absorption of a single photon results in two or more electron–hole pairs. Quantum dots are promising materials for implementing carrier multiplication principles in real-life technologies. So far, however, most of research in this area has focused on optical studies of solution samples with yet to be proven relevance to practical devices. Here we report ultrafast electro-optical studies of device-grade films of electronically coupled quantum dots that allow us to observe multiplication directly in the photocurrent. Our studies help rationalize previous results from both optical spectroscopy and steady-state photocurrent measurements and also provide new insights into effects of electric field and ligand treatments on multiexciton yields. Importantly, we demonstrate that using appropriate chemical treatments of the films, extra charges produced by carrier multiplication can be extracted from the quantum dots before they are lost to Auger recombination and hence can contribute to photocurrent of practical devices.

## Abstract (web summary)

In semiconductors, the absorption of a high energy photon can result in the formation of several charge pairs. Here the authors perform ultrafast photocurrent measurements on thin films to explore how quantum dot couplings and the electric field influence multiexciton photovoltaic devices.

## Graphical abstract

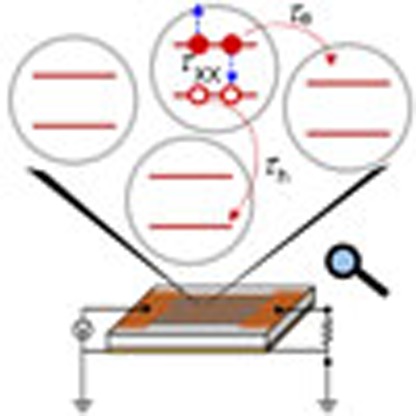

Processes of photon-to-charge-carrier conversion are of key importance in photosynthesis, generation of solar fuels, and production of photoelectricity. A traditional assumption is that a single photon is converted into a single electron–hole (e–h) pair or an exciton. However, the fundamental laws of physics do not prohibit the absorption of a single photon from producing multiple e–h pairs if the photon energy (*hv*) is sufficiently high to ensure energy conservation. This process, usually termed ‘carrier multiplication' (CM) or ‘multiexciton generation', can be understood in terms of impact ionization whereby a valence band electron is promoted across a band gap (*E*_g_) as a result of a collision with an energetic carrier (an electron or hole) (Fig. 1a). Ideally, quantum efficiency (QE) of photon-to-exciton conversion (*q*) in the presence of CM is characterized by a staircase-like function where each increment of *hv* by *E*_g_ results in a new e–h pair, that is, an increase in QE by 100% (Fig. 1b; blue line). If CM operates at this energy-conservation defined limit, it could lead to dramatic advances in solar energy conversion technologies^1^,^2^.

In macroscopic, ‘bulk' semiconductors, however, CM is inefficient within the solar spectrum due to a high activation threshold (*E*_CM_) which is at least ∼4*E*_g_ (refs 3, 4) (Fig. 1b; red line). As was originally demonstrated in ref. 5 the CM threshold could be considerably reduced (to <3*E*_g_) using nanosized semiconductor crystals known as quantum dots (QDs), and further pushed down to the fundamental 2*E*_g_ limit through QD hetero-structuring^6^ (Fig. 1b; green line). The observed reduction in *E*_CM_ can be linked to relaxation of constrains imposed by translation momentum conservation due to suppression of translation motion of carriers in QDs^7^,^8^,^9^. In addition, CM in QDs benefits from a discrete, atomic-like structure of electronic states, which makes a competing process of photon emission less efficient than in the bulk^2^. Following the original report on CM in PbSe QDs^5^, this process has been observed in QDs of a variety of compositions^5^,^10^,^11^,^12^,^13^,^14^,^15^, as well as other nanomaterials including semiconductor nanorods^16^, semiconductor hetero-structures^6^,^17^, carbon nanotubes^18^ and graphene^19^. Considerable progress has also been made towards demonstrating CM-enhanced photocurrent in practical devices^20^,^21^,^22^ as well as the development of various theoretical frameworks for treating this process in nanomaterials^8^,^23^,^24^,^25^,^26^,^27^,^28^.

While the conducted studies have indicated a significant promise of engineered nanostructures for obtaining enhanced CM, there are still a number of questions and challenges that need to be addressed to fully realize the potential of CM in practical devices. For example, the majority of quantitative insights into CM has been derived from optical spectroscopic studies of solutions of isolated QDs^29^,^30^,^31^, while practical devices use films of electronically coupled particles^20^,^22^,^32^,^33^,^34^,^35^. The available studies of QD films, however, do not provide a conclusive answer to the question on the effect of electronic coupling on multiexciton yields. For example, time-resolved microwave conductivity measurements indicate that CM efficiencies in PbSe QD films can be considerably higher than those in QD solutions^36^. On the other hand, the CM yields obtained from studies of PbSe QD-based solar cells are lower than those from ultrafast spectroscopic studies of solution samples^22^. Therefore, it is still unclear how the CM yields determined spectroscopically for QD solutions translate into those observable in photocurrent in photoconducting films. A related problem is a still unknown effect of electric field on CM, which is an important factor in *p*–*n* junction devices. Yet another open question is the role of nonradaitive multicarrier Auger decay. This process is inherent to CM-enabled devices and owing to its extremely short timescales^37^ it can potentially erase all gains due to CM. An outstanding challenge is also the lack of experimental means for rapid and reliable evaluation of CM performance of nanomaterials. Ultrafast spectroscopic techniques, applied most commonly in CM measurements, use complex and expensive equipment, are labor-intense and very time consuming, which is a serious obstacle to more rapid progress in the development of new, more efficient CM materials.

In the present report we demonstrate that the above challenges can be successfully addressed by using an ultrafast transient photocurrent (TPC) technique for studies of early time electronic dynamics, and specifically CM, in device-grade films of coupled QDs. This method has been previously applied to, for example, organic materials^38^,^39^, however, up to now not to QDs. In our studies, we incorporate QD films into a fast electro-optical switch^40^ triggered by femtosecond laser pulses and monitor dynamics of photogenerated carriers with 40 ps resolution which allows us to resolve Auger decay of multicarrier states directly in photocurrent. Following a rigorous validation of this method via a quantitative analysis of TPC signatures of single excitons and biexcitons, we use it to investigate CM yields in QD films and then compare them with existing literature on optical studies of QD solutions and photocurrent-based measurements of QD devices. Application of TPC helps rationalize these previous results and also provides new insights into the effects of an electric field and ligand treatments on CM yields as well as competition between Auger recombination and charge extraction from the QDs.

## Results

### TPC technique

**Figure 2:**
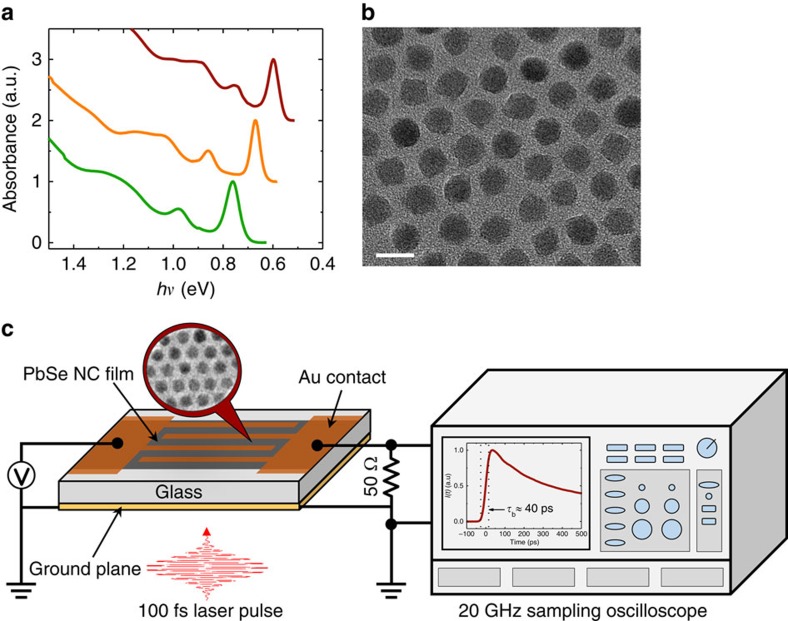
Schematic of TPC measurements. (**a**) Absorption spectra of QDs of three different mean radii of 2.8 nm (bottom), 3.0 nm (middle) and 3.5 nm (top). (**b**) An example of a transmission electron microscopy image of PbSe QDs with a mean radius of 3.5 nm, scale bar is 10 nm. (**c**). A photoconductive switch comprises a thin film of PbSe QDs deposited on a glass substrate with top interdigitated Au contacts. Photocurrent is excited by short ∼100 fs laser pulses and monitored with a 20 GHz sampling oscilloscope. An example of a TPC trace on the oscilloscope's screen shows a ca. 40 ps buildup (*τ*_b_) corresponding to the time for the signal to increase from 10 to 90% of the peak amplitude; this constitutes the overall temporal resolution of the system.

In these studies, we use PbSe QDs with mean radii (*R*) from 2.8 to 3.5 nm (Fig. 2a,b) and corresponding band gaps of 0.60–0.76 eV as inferred from the position of the band-edge peak in optical absorption spectra (Fig. 2a). The dots are incorporated into a photoconductive switch (Fig. 2c), which comprises a ∼200-nm thick QD layer assembled on a glass substrate with a 100 nm-thick gold ground plane on its back side. The QDs are treated with 1,2-ethanedithiol (EDT) or EDT/hydrazine using procedures that are similar to those applied in previous studies of charge transport in QD films^41^,^42^,^43^,^44^, QD-based photodetectors^20^,^35^ and solar cells^22^. The device is completed by thermally evaporating 100 nm thickness interdigitated gold contacts prepared as co-planar microstrips with a 50 Ω impedance. One contact of the switch is biased with an adjustable d.c. voltage, while the other is connected to the input port of a fast sampling oscilloscope with a 20 GHz bandwidth. The switch is triggered with short ∼100 fs laser pulses of an amplified Ti:sapphire laser at 1.55 eV (fundamental output) or 3.1 eV (frequency doubled output). We characterize the excitation intensity in terms of the average number of photons absorbed per QD per pulse <*N*_abs_> which can be expressed via a per-pulse photon fluence (*w*) and an absorption cross section (*σ*) as <*N*_abs_>=*wσ*. The overall system response time is ∼40 ps as illustrated by the resolution-limited signal rise time in Fig. 2c. All measurements reported below have been conducted at room temperature.

Temporal evolution of a photocurrent across the switch can be described by the following expression^40^: 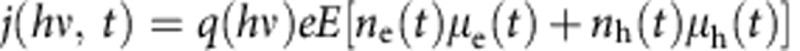, where *j*(*hv*, *t*) is the current density which is directly proportional to the experimentally measured photocurrent *I*(*t*), *e* is the electron charge, *E* is the applied electric field, *n*_e_ and *n*_h_ are the densities of photogenerated electrons and holes, respectively, and *μ*_e_ and *μ*_h_ are their mobilities. Expressing *n*_e_ and *n*_h_ in terms of the density of the QDs (*n*_QD_) and QD average electron and hole occupancies (<*N*_e_> and <*N*_h_>, respectively), and further assuming that because of mirror-symmetric conduction and valence bands of PbSe electron and hole mobilities are similar (*μ*_e_=*μ*_h_=*μ*), we can present *j* as 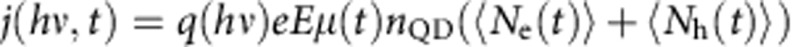. If we further assume that on experimental time scales (*t*<3 ns) the QD occupancies change only as a result of e–h recombination and thus at all times <*N*_e_>=<*N*_h_>=<*N*>, we arrive at the following equation:


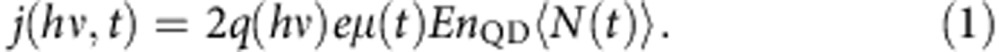


Equation (1) suggests that in general the dynamics of the measured photocurrent is governed by temporal evolutions of both mobilities and QD occupancies. However, if the changes in mobility due to, for example, charge trapping at intraband defects are slow compared with recombination time scales, the photocurrent directly reports on carrier population dynamics. In this regime, TPC is not sensitive to exciton dissociation or charge migration between the dots as these processes do not affect the average occupancy of the QDs. Equation (1) also suggests that the contribution from a given charge to photocurrent does not dependent on the total number of other charges residing in the QD, meaning in particular that the contribution from a biexciton to *j* is twice that of a single exciton, as confirmed below based on the analysis of TPC data. The listed features of the TPC technique have much in common with those of transient absorption, especially when it is used to monitor the band-edge bleach, as it's also proportional to the average occupancy of the QDs^45^. As a result, as we show below, some of the methods developed for the analysis of transient absorption data can be directly applied to TPC measurements.

### Validation studies using sub-CM-threshold excitation

**Figure 3:**
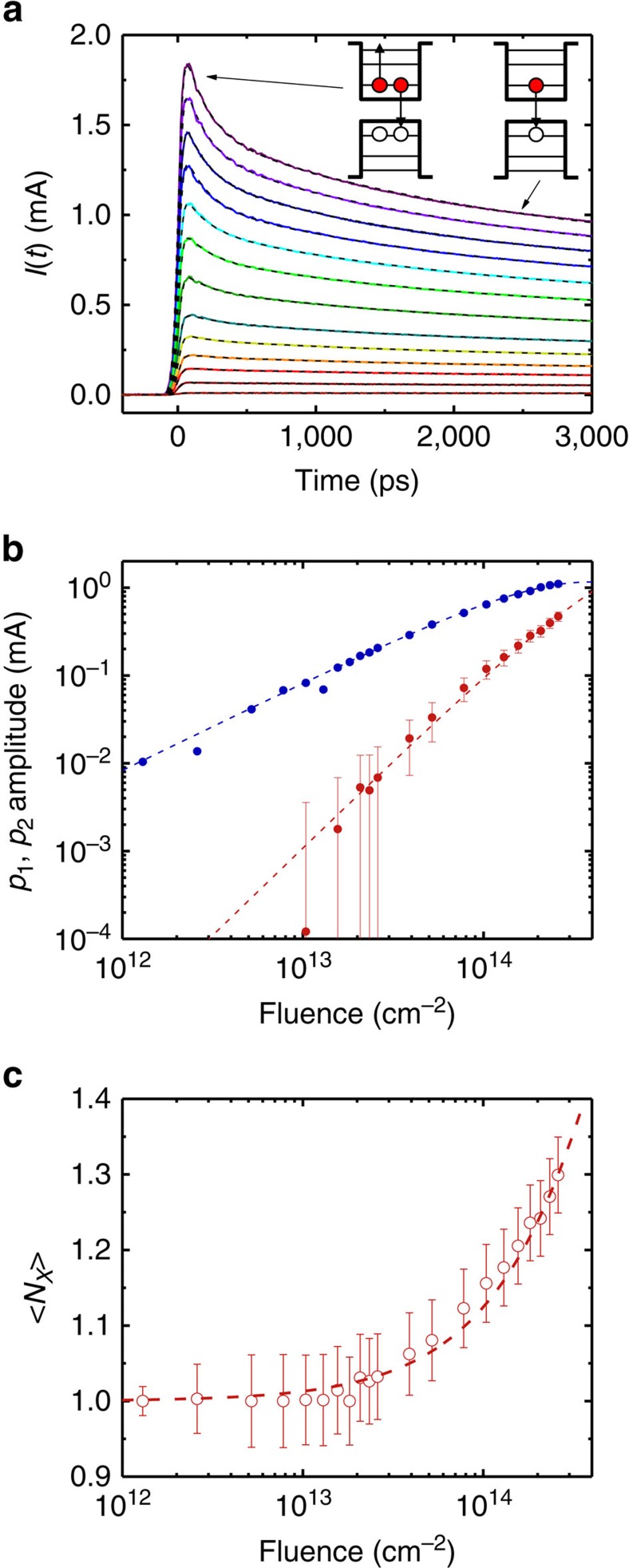
TPC measurements using excitation with a 1.55 eV sub-CM-threshold photon energy. (**a**) Pump-power dependence of TPC traces measured for the EDT-treated film of QDs with *E*_g_=0.69 eV for <*N*_abs_> varied from 0.003 to 0.68 (from bottom to top; coloured lines); the applied bias *V*=60 V. Increasing fluence leads to the development of a fast component consistent with multicarrier Auger recombination. Fits to the data are shown as the dashed black lines; see text for details of the fitting procedure. (**b**) The pump-fluence dependence of fractions of single excitons (*p*_1_, blue circles) and biexcitons (*p*_2_, red squares) generated in a QD ensemble. The data can be accurately described assuming Poisson statistics of carrier distributions (lines). (**c**) Exciton multiplicities calculated based on the measured *p*_1_ and *p*_2_ (circles) are perfectly described by the Poisson dependence (dashed line) and indicate that <*N*_X_> tends to unity in the limit of zero fluences, as expected for the no-CM case. Error bars in **b** and **c** are s.d. derived from the non-linear least squares fits to the measured photocurrent transients used to derive amplitudes of the single-exciton and multiexciton decay components.

In Fig. 3a, we display TPC data for EDT-treated QD films obtained using excitation at 1.55 eV and an electrical bias *V*=60 V. The QD mean radius is ∼3.5 nm and the band gap is 0.69 eV, which corresponds to *hν*=2.25*E*_g_. The CM threshold for PbSe QDs is ∼2.7–2.8*E*_g_ (ref. 29), so we expect to find no signatures of CM for this photon energy. At low fluences when <*N*_abs_> <<1, the TPC shows fairly slow dynamics that are virtually independent on pump intensity (that is, differ only by a constant multiplier), as expected for the situation when QDs are excited with single excitons and the increase in <*N*_abs_> leads only to the increase in the number of photoexcted QDs. These single-excitonic dynamics can be closely described by an exponential decay with a constant offset: 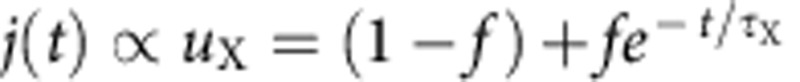, where *τ*_X_=1.7±0.1 ns and *f*=0.6±0.1.

When the excitation fluence is increased (<*N*_abs_> becomes ∼0.5 and higher), the measured transients develop a short early time component, which is a typical signature of multiexcitons decaying via an Auger process whereby the e–h recombination energy is transferred to the third carrier. According to a well-established volume scaling^37^, the biexciton Auger lifetime (*τ*_A,XX_) in 3.5 nm radius QDs is expected to be ∼180 ps, and this is very close to the 170±10 ps time constant of the early time TPC component. A similar agreement between the measured decay and expectations based on either volume scaling or literature results is also observed for all other studied QD sizes (see Supplementary Fig. 1). This confirms the interpretation of the early time TPC component in terms of the biexciton Auger decay, which we describe by 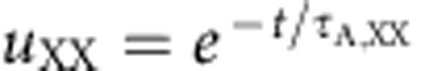.

For the quantitative analysis of the measured transients we consider the situation of moderate pump levels when excitation produces only single excitons and biexcitons with fractions *p*_1_ and *p*_2_ within the QD ensemble. In the case of linear scaling of *j* with *N* (see equation (1)), the early time photocurrent can be expressed as *j*(*t*=0)∝*p*_1_+2*p*_2_. During Auger decay all biexcitons are converted into single exciton and if *τ*_A,XX_<<*τ*_X_ (as indicated by our measurements; see above) the fraction of singly excited QDs becomes equal to (*p*_1_+*p*_2_), which yields *j*(*τ*_A,XX_<*t*<*τ*_X_)∝*p*_1_+*p*_2_. On the basis of these prior- and post-Auger-decay conditions, we can present the overall temporal evolution of the photocurrent as (see Supplementary Note 1 for a detailed derivation of this expression):


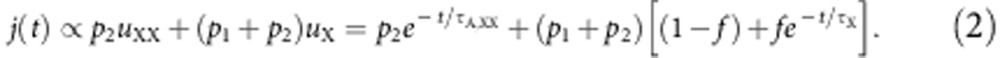


Now, we can determine fractions of single excitons and biexciton in a photogenerated ensemble by fitting TPC traces from Fig. 3a to equation (2) with *p*_1_ in and *p*_2_ as adjustable parameters. The fits are additionally convoluted with a Gaussian to account for the finite temporal resolution (see Supplementary Note 1 and Supplementary Fig. 2 for additional information). The values of *p*_1_ in and *p*_2_ derived from this procedure are plotted in Fig. 3b as a function of *w*.

To validate the derivation of *p*_1_ in and *p*_2_, we conduct a statistical analysis of a carrier distribution across a QD ensemble. In the absence of CM, it is expected to follow Poisson statistics defined by a single parameter, which is the average QD occupancy <*N*(*t*=0)>=<*N*_abs_>=*wσ* (ref. 45). Calculating *p*_1_ in and *p*_2_ on the basis of Poisson distribution (see Supplementary Note 2 for further details), and using *σ* and a shared amplitude factor as the two adjustable parameters we can simultaneously fit the pump-intensity dependence of *p*_1_ in and *p*_2_. On the basis of the fitting, *σ* is 2.6 × 10^−15^ cm^2^, which is in close agreement with the estimation based on the QD volume^45^ as well as published literature values^46^. Absorption cross-sections derived for other QD sizes from a similar Poissonian analysis of TPC data are also consistent with values documented in the literature (Supplementary Fig. 1). This confirms that our TPC measurements indeed allow us to quantify relative fractions of single excitons and biexcitons, which is a key capability in CM studies.

Next, we evaluate average exciton multiplicity, <*N*_X_>, which is the average number of e–h pairs per photoexcited QD. The zero-pump-fluence limit of this quantity defines the QE of photon-to-exciton conversion: *q*=lim_*w*→0_<*N*_X_> (ref. 5). For a photoexcited system considered here, which contains only single excitons and biexcitons,


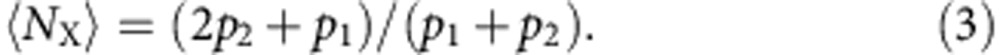


Using *p*_1_ and *p*_2_ derived from the TPC traces, we calculate <*N*_X_> as a function *w* and plot the results in Fig. 3c (red circles). As expected for the situation with no CM, <*N*_X_>→1 in the limit of *w*→0. Again, we observe a close agreement between the measured exciton multiplicities and those calculated based on the Poisson analysis (Fig. 3c, red line) using the same cross section as derived from the fit in Fig. 3b.

### CM studies of QD films

**Figure 4:**
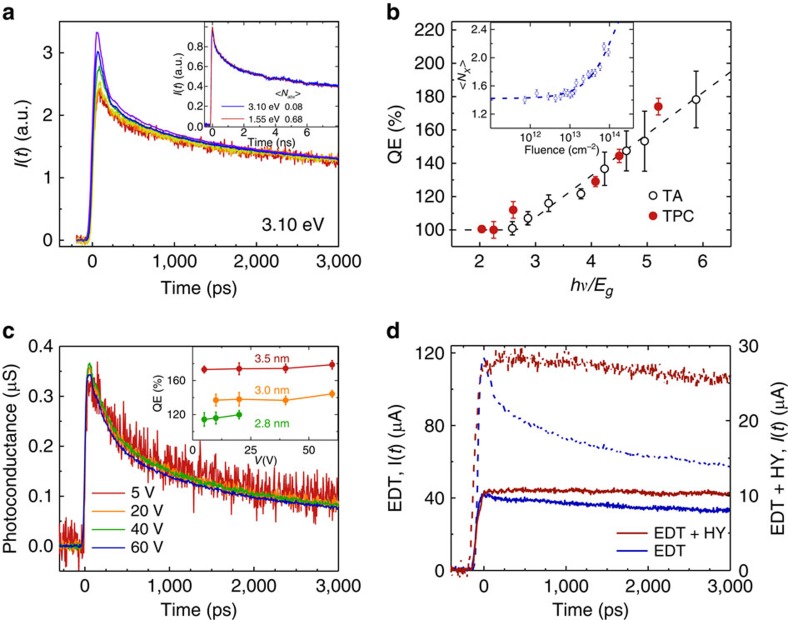
TPC measurements of CM using excitation with 3.1 eV photons. (**a**) Tail-normalized TPC traces with excitation above the CM threshold show the persistence of a fast Auger decay component in the limit of low pump fluences, indicating the presence of biexcitons generated via CM (same sample as in Fig. 3; same bias); <*N*_abs_> varies from 0.04 to 1.3 (from bottom to top). The inset shows that the low-fluence TPC trace measured with 3.1 eV photons can be reproduced using 1.5 eV excitation but with a much higher pump intensity. (**b**) Comparison of the CM yields from the present TPC measurements (red solid circles) to those from previous transient absorption experiments on colloidal suspensions (open black circles; ref. 48). Error bars shown for the TPC data points are s.d. derived from the non-linear least squares fits to the photocurrent transients used in the evaluation of CM yields. Inset: derivation of the CM yield from the low-pump-intensity limit of the measured multiplicities. (**c**) The photoconductance for the same sample as in Fig. 3 excited with 3.1 eV photons with <*N*_abs_>=0.04 exhibits Ohmic dependence, indicating no influence of the electric field on the CM yield. The inset shows that the CM yield is virtually electric-field independent for all QD sizes studied. Error bars are derived in the same way as in **b**. (**d**) The QD film treated with EDT/hydrazine (red) maintains the same increase in the early time signal amplitude as the EDT-treated film (black) when comparing 3.1 eV (dashed) and 1.55 eV (solid) excitation. However, in the case of the EDT/hydrazine treatment, the 3.1 eV trace lacks initial Auger decay indicating that photogenerated carriers escape from the dot before they undergo Auger recombination. Both films are comprised of QDs with a band gap of 0.69 eV and an electrical bias of 60 V is used. For the EDT- and EDT/hydrazine-treated films the fluences are, respectively, ∼7 × 10^12^ and ∼2 × 10^11^ cm^−2^ for both excitation energies.

To study CM, we use pump pulses at 3.1 eV. In the example in Fig. 4a we apply them to the sample with *E*_g_=0.69 eV (same as in Fig. 3), which corresponds to *hν*/*E*_g_=4.5. At high excitation fluences, we again observe the fast decay component due to Auger recombination of biexcitons. However, in contrast to the 1.55 eV excitation, this component persists in the limit of extremely low excitation intensities (down to <*N*_abs_> of 0.007) when the likelihood of multiple photon absorption is negligibly small (<1%). This implies that biexcitons are generated by single photons, that is, are due to CM. Using the fitting procedure described above we obtain the exciton multiplicity and plot it in the inset of Fig. 4b as a function of *w*. By extrapolating experimental data to zero excitation fluence we obtain QE=1.44±0.04 (144%), and further multiexciton yield *η*=(*q*–1) of 0.44±0.04 or 44%. We conduct similar measurements for samples with other QD sizes and summarize all results in Fig. 4b (red circles).

Interestingly, we can replicate the low-pump-intensity 3.1 eV TPC traces using 1.5 eV excitation, however, with much higher pump fluences. As illustrated in the example of Fig. 4a inset, the 3.1 eV trace taken with <*N*_abs_>=0.08 perfectly matches the 1.5 eV traces acquired with <*N*_abs_>=0.68, implying identical exciton multiplicities. As we showed earlier, for 1.5 eV excitation, <*N*_X_> is controlled by Poisson distribution uniquely defined by <*N*_abs_>. The fact that in the case of 3.1 eV excitation the same value of <*N*_X_> is realized for much lower fluence indicates that carrier distribution across the QD ensemble is non-Poissonian, which is a distinct attribute of the CM process^47^.

### Comparison with QD solutions

As was mentioned earlier, an important and still open question is the relationship between CM yields observed spectroscopically for QD solutions and those attainable in devices usimg coupled QD films. Compared with steady state or ‘slow' time-resolved techniques used in previous measurements of QD films^22^,^36^, the TPC method is a more suitable tool for assessing the CM performance of film versus solution samples as it probes CM under conditions that largely replicate ultrafast optical measurements and thus allow for elimination of potential distortions in CM yields due to recombination and/or transport carrier losses. In Fig. 4b, we compare our TPC results with CM measurements from previous ultrafast optical studies of PbSe QD solutions^48^. For all studied QD sizes, we find a close agreement between these two sets of data, which leads us to an important conclusion that CM yields observed in QD solutions can in principle be reproduced in QD-film-based devices, at least at early stages after photoexcitation before any significant carrier losses to recombination occur. This also implies that a large body of existing spectroscopic data on CM in various classes of QDs can be used for evaluating their prospective performance in devices.

In addition to the advantage of replicating ultrafast optical measurements in a more device-relevant regime, the TPC method also allows for a significant time saving in evaluating CM performance of materials. For example, even at the lowest pump fluences (<*N*_abs_> <0.01), it takes <1 min to acquire a low-noise TPC trace and <1 h to obtain a CM yield from several TPC measurements conducted at different pump powers. On the other hand, it would require more than 12 h to record a single trace with a comparable signal-to-noise ratio with standard transient absorption or PL techniques (for a considerably smaller number of data points), and a few days to complete a single CM-yield measurement.

### Effect of electric field

An interesting opportunity opened by the TPC technique is the studies of CM under conditions not accessible with standard spectroscopic methods applied to QD solutions. For example, using TPC we can readily evaluate the effect of an applied electric field on CM yields. It is known that impact ionization, which underlies CM in extended bulk solids, is enhanced by electric field^49^, however, its effect on CM in QDs has never been evaluated either experimentally or theoretically. To fill this gap, here we analyse multiexciton yields as a function of bias applied to a photoconductive switch in the range up to 60 V (*E* up to ∼24 kV cm^−1^). Control experiments conducted below the CM threshold (1.55 eV excitation) at low fluence where Auger decay is not present indicate that the photoconductance (*I*(*t*)/*V*) is constant, indicating Ohmic behaviour (Supplementary Fig. 3). Changing the excitation to above the CM threshold we again observe no dependence of measured dynamics on applied voltage apart from a linear increase of photocurrent with *V* (Fig. 4c). This behaviour, observed for all studied QD sizes (Fig. 4c inset), indicates that the CM yield is *E*-independent, at least in the range of biases used in these measurements. Further, since applied fields are of the order of those existing in a depletion layer of *p*–*n* junction QD solar cells^50^, we can conclude that the presence of built-in electric fields in practical photovoltaic structures should not affect the CM performance of the QDs.

### Interplay between Auger recombination and charge extraction

A high temporal resolution of TPC measurements also allows us to evaluate the influence of fast multicarrier Auger recombination on performance of practical devices. While in standard solar cells it is only active at high light intensities (for example, under concentrated solar radiation), it's always present in CM-based devices independent on incident flux and thus can potentially eliminate all potential photocurrent gains if charge extraction is not sufficiently fast. For example, in EDT-treated QD films in Figs 3 and 4, we clearly resolve fast photocurrent decay due to Auger recombination. While this prominent feature helps us detect CM and quantify its yield, it also tells us that all extra charges generated through CM are lost to non-radiative recombination before extraction. This result helps rationalize the observations of ref. 22 that EDT-treated films did not exhibit any boost in steady-state photocurrent due to CM. On the basis of our TPC measurements, the lack of CM-enhancement in this case was primarily due to early time carrier losses to Auger recombination but not, for example, charge transport losses or exciton quenching by electrodes.

The above considerations point to an important role of inter-dot coupling in CM-based devices that must be sufficiently strong to ensure fast charge extraction outpacing Auger decay. Mild hydrazine treatment following the treatment with EDT is known to enhance inter-dot coupling and also to improve passivation of surface defects^44^. When we apply a combined EDT–hydrazine treatment to our QD films and then measure them using sub-CM-threshold 1.5 eV excitation, we do observe suppression of defect related relaxation as indicated by nearly decay free TPC traces on a nanosecond timescale (Fig. 4d). Interestingly, when we switch to 3.1 eV excitation, which is above the CM threshold, we do not observe signatures of Auger decay at low fluence either, while they were present in EDT-treated film. Furthermore, the increase in the early time signal amplitude between 1.55 and 3.1 eV, observed for the same incident fluence, is identical for both EDT and hydrazine treated films, indicating that the CM yield was not diminished with the treatment process. Using equation (1) we may estimate the CM yield in the EDT–hydrazine-treated films based on the peak photocurrent value as 148%, which is in agreement with the 144±4% yield found for the EDT-treated films (see Supplementary Note 3 for additional details). This indicates that the applied surface treatment does improve inter-dot coupling such as separation of multiple charges between adjacent QDs occurs faster than Auger recombination but does not change the number of additional charges generated through CM. This result is again in agreement with previous steady-state photocurrent studies of ref. 22 according to which QD films treated with a combination of EDT and hydrazine show clear signatures of CM as manifested in more than 100% external QEs in extracted photocurrent. Together with earlier measurements our newest ultrafast photocurrent data affirm a high potential of CM for boosting photocurrent of practical devices.

## Methods

### PbSe QD synthesis

The PbSe QD synthesis and Cl passivation were adapted from a previously reported method^51^. All syntheses and manipulations were performed under dry argon using standard Schlenk-line and glove-box techniques. Oleic acid (90%), lead (II) oxide (PbO, 99.999%), selenium shot (Se, 99.999%), 1-octadecene (90%), Bis(trimethylsilyl)sulfide (TMS_2_S, 95%), di-*i*-butylphosphine ((*i*-Bu)_2_PH, 98%) and trioctylphosphine (TOP, 97%) were used without additional purification. PbO (1.1 g), Oleic acid (5 ml) and 1-octadecene (20 ml) were heated to 120 °C under vacuum for 30 min, then the solution was purge with argon and heated to 180 °C. A volume of 5 ml of 2 M trioctylphosphine selenide (TOPSe) and ∼50 μl of ((*i*-Bu)_2_PH were rapidly injected, and the solution was kept at 160 °C for 1–10 min, depending on desired QD size. PbSe QDs were precipitated with excess ethanol and centrifugation to remove the decantate, and then redispersed in hexane. To passivate the QD surface with Cl, aqueous hydrochloric acid (37%) and sodium hypochlorite (6%) were mixed to generate Cl_2_. The resultant aqueous solution was mixed with CCl_4_ that subsequently phase separates into an aqueous and organic layer, with the Cl_2_ extracted into the CCl_4_ layer. After taking CCl_4_ layer, the solution was dried by anhydrous CaCl_2_. QD solution and Cl_2_/CCl_4_ solution were mixed, and Cl-treated QDs were purified by adding excess ethanol and centrifugation to remove decantate. The final QDs were redispersed in hexane.

### Fabrication of devices and their characterization

A PbSe QD film was deposited onto a glass substrate with a prefabricated 100 nm-thick gold ground plate on its back side in a layer-by-layer fashion to a film thickness of ∼200 nm (ref. 52). The 100 nm thickness co-planar Au microstrips of 50 Ω impedance were then thermally deposited on top of the QD film under a vacuum of 2 × 10^−6^ Torr. The gap between the gold microstrips was 25 μm. One side contact of the device was biased with a tunable voltage d.c. power supply, while the other side contact was connected to a 20 GHz bandwidth oscilloscope through coaxial cables. The oscilloscope was triggered by laser pulses using a high-speed Si photodetector to minimize timing jitter. A regeneratively amplified Ti:sapphire laser producing ∼100 fs pulses at 1.55 or 3.1 eV with a repetition rate of 250 kHz was used as an excitation source. The laser beam was focused with a cylindrical achromatic lens into a line of a ∼100–300 μm width, which produced spatially uniform excitation across the photoactive area of a device. The system response time was ∼40 ps. All devices were fabricated in a nitrogen glove box and sealed in an argon-filled housing for measurements to avoid oxidation of the samples.

## Additional information

**How to cite this article:** Gao, J. *et al*. Carrier multiplication detected through transient photocurrent in device-grade films of lead selenide quantum dots. *Nat. Commun.* 6:8185 doi: 10.1038/ncomms9185 (2015).

## Supplementary Material

Supplementary Information Supplementary Figures 1-3, Supplementary Notes 1-3 and Supplementary References.

## Figures and Tables

**Figure 1:**
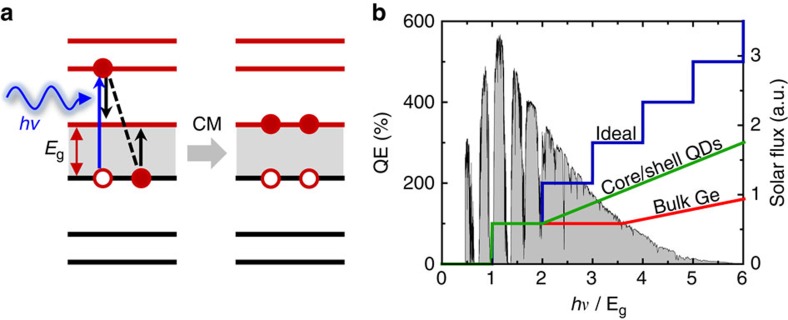
CM and its characteristics. (**a**) Absorption of a photon with a sufficiently high energy (*hv*) generates a hot carrier, which can excite an additional e–h pair via an impact-ionization-like collision with a valence band electron. As a result, two e–h pairs are produced per single absorbed photon. (**b**) Comparison of the ideal QE of photon-to-exciton conversion in the energy-conserving limit (blue) with those of bulk germanium (red) and PbSe/CdSe core/shell QDs (green) assuming the same band gap of 0.67 eV, which is equal to *E*_g_ of Ge; the Ge and QD data are adopted from refs 4, 6, respectively. Owing to the reduced threshold (*E*_CM_), CM in nanomaterials is active over a wider fraction of the solar spectrum (grey shading) than in the bulk.

## References

[b1] Werner J. H., Kolodinski S. & Queisser H. J. Novel optimization principles and efficiency limits for semiconductor solar cells. Phys. Rev. Lett. 72, 3851–3854 (1994). 10056313 10.1103/PhysRevLett.72.3851

[b2] Nozik A. J. Quantum dot solar cells. Phys. E 14, 115–120 (2002).

[b3] Alig R. C. & Bloom S. Electron-hole-pair creation energies in semiconductors. Phys. Rev. Lett. 35, 1522–1525 (1975).

[b4] Koc S. The quantum efficiency of the photo-electric effect in germanium for the 0.3–2 μ wavelength region. Czech. J. Phys. 7, 91–95 (1957).

[b5] Schaller R. D. & Klimov V. I. High efficiency carrier multiplication in PbSe nanocrystals: Implications for solar energy conversion. Phys. Rev. Lett. 92, 186601 (2004). 15169518 10.1103/PhysRevLett.92.186601

[b6] Cirloganu C. M. . Enhanced carrier multiplication in engineered quasi-type-II quantum dots. Nat. Commun. 5, 4148 (2014). 24938462 10.1038/ncomms5148 PMC4083434

[b7] Pietryga J. M., Zhuravlev K. K., Whitehead M., Klimov V. I. & Schaller R. D. Evidence for barrierless Auger recombination in PbSe nanocrystals: a pressure-dependent study of transient optical absorption. Phys. Rev. Lett. 101, 217401 (2008). 19113449 10.1103/PhysRevLett.101.217401

[b8] Shabaev A., Efros A. L. & Nozik A. J. Multiexciton generation by a single photon in nanocrystals. Nano Lett. 6, 2856–2863 (2006). 17163719 10.1021/nl062059v

[b9] Rupasov V. I. & Klimov V. I. Carrier multiplication in semiconductor nanocrystals via intraband optical transitions involving virtual biexciton states. Phys. Rev. B 76, 125321 (2007).

[b10] Murphy J. E. . PbTe colloidal nanocrystals: synthesis, characterization, and multiple exciton generation. J. Am. Chem. Soc. 128, 3241–3247 (2006). 16522105 10.1021/ja0574973

[b11] Schaller R. D., Pietryga J. M. & Klimov V. I. Carrier multiplication in InAs nanocrystal quantum dots with an onset defined by the energy conservation limit. Nano Lett. 7, 3469–3476 (2007). 17967043 10.1021/nl072046x

[b12] Beard M. C. . Multiple exciton generation in colloidal silicon nanocrystals. Nano Lett. 7, 2506–2512 (2007). 17645368 10.1021/nl071486l

[b13] Kobayashi Y., Udagawa T. & Tamai N. Carrier multiplication in CdTe quantum dots by single-photon timing spectroscopy. Chem. Lett. 38, 830–831 (2009).

[b14] Stubbs S. K. . Efficient carrier multiplication in InP nanoparticles. Phys. Rev. B 81, 081303 (2010).

[b15] Trinh M. T. . Direct generation of multiple excitons in adjacent silicon nanocrystals revealed by induced absorption. Nat. Photonics 6, 316–321 (2012).

[b16] Padilha L. A. . Aspect ratio dependence of Auger recombination and carrier multiplication in PbSe nanorods. Nano Lett. 13, 1092–1099 (2013). 23360573 10.1021/nl304426y

[b17] Gachet D., Avidan A., Pinkas I. & Oron D. An upper bound to carrier multiplication efficiency in type II colloidal quantum dots. Nano Lett. 10, 164–170 (2009). 19911830 10.1021/nl903172f

[b18] Wang S. J., Khafizov M., Tu X. M., Zheng M. & Krauss T. D. Multiple exciton generation in single-walled carbon nanotubes. Nano Lett. 10, 2381–2386 (2010). 20507082 10.1021/nl100343j

[b19] Plötzing T. . Experimental verification of carrier multiplication in graphene. Nano Lett. 14, 5371–5375 (2014). 25144320 10.1021/nl502114w

[b20] Sukhovatkin V., Hinds S., Brzozowski L. & Sargent E. H. Colloidal quantum-dot photodetectors exploiting multiexciton generation. Science 324, 1542–1544 (2009). 19541992 10.1126/science.1173812

[b21] Sambur J. B., Novet T. & Parkinson B. A. Multiple exciton collection in a sensitized photovoltaic system. Science 330, 63–66 (2010). 20929804 10.1126/science.1191462

[b22] Semonin O. E. . Peak external photocurrent quantum efficiency exceeding 100% via MEG in a quantum dot solar cell. Science 334, 1530–1533 (2011). 22174246 10.1126/science.1209845

[b23] Witzel W. M., Shabaev A., Hellberg C. S., Jacobs V. L. & Efros A. L. Quantum simulation of multiple-exciton generation in a nanocrystal by a single photon. Phys. Rev. Lett. 105, 137401 (2010). 21230809 10.1103/PhysRevLett.105.137401

[b24] Gali A., Vörös M., Rocca D., Zimanyi G. T. & Galli G. High-energy excitations in silicon nanoparticles. Nano Lett. 9, 3780–3785 (2009). 19785388 10.1021/nl901970u

[b25] Allan G. & Delerue C. Role of impact ionization in multiple exciton generation in PbSe nanocrystals. Phys. Rev. B 73, 205423 (2006).

[b26] Jaeger H. M., Hyeon-Deuk K. & Prezhdo O. V. Exciton multiplication from first principles. Acc. Chem. Res. 46, 1280–1289 (2013). 23459543 10.1021/ar3002365

[b27] Rabani E. & Baer R. Theory of multiexciton generation in semiconductor nanocrystals. Chem. Phys. Lett. 496, 227–235 (2010).

[b28] Califano M., Zunger A. & Franceschetti A. Direct carrier multiplication due to inverse Auger scattering in CdSe quantum dots. Appl. Phys. Lett. 84, 2409–2411 (2004).

[b29] Klimov V. I. Multicarrier interactions in semiconductor nanocrystals in relation to the phenomena of Auger recombination and carrier multiplication. Ann. Rev. Cond. Mat. Phys. 5, 285–316 (2014).

[b30] Ten Cate S. . Generating free charges by carrier multiplication in quantum dots for highly efficient photovoltaics. Acc. Chem. Res. 48, 174–181 (2015). 25607377 10.1021/ar500248g

[b31] Beard M. C., Luther J. M., Semonin O. E. & Nozik A. J. Third generation photovoltaics based on multiple exciton generation in quantum confined semiconductors. Acc. Chem. Res. 46, 1252–1260 (2012). 23113604 10.1021/ar3001958

[b32] Kamat P. V. Quantum dot solar cells. Semiconductor nanocrystals as light harvesters†. J. Phys. Chem. C 112, 18737–18753 (2008).

[b33] Coe S., Woo W.-K., Bawendi M. G. & Bulovic V. Electroluminescence from single monolayers of nanocrystals in molecular organic devices. Nature 420, 800–803 (2002). 12490945 10.1038/nature01217

[b34] Luther J. M. . Schottky solar cells based on colloidal nanocrystal films. Nano Lett. 8, 3488–3492 (2008). 18729414 10.1021/nl802476m

[b35] Konstantatos G. . Ultrasensitive solution-cast quantum dot photodetectors. Nature 442, 180–183 (2006). 16838017 10.1038/nature04855

[b36] Sandeep C. S. S. . High charge-carrier mobility enables exploitation of carrier multiplication in quantum-dot films. Nat. Commun. 4, 2360 (2013). 23974282 10.1038/ncomms3360 PMC3759061

[b37] Klimov V. I., Mikhailovsky A. A., McBranch D. W., Leatherdale C. A. & Bawendi M. G. Quantization of multiparticle Auger rates in semiconductor quantum dots. Science 287, 1011–1013 (2000). 10669406 10.1126/science.287.5455.1011

[b38] Moses D., Soci C., Chi X. & Ramirez A. P. Mechanism of carrier photogeneration and carrier transport in molecular crystal tetracene. Phys. Rev. Lett. 97, 067401 (2006). 17026202 10.1103/PhysRevLett.97.067401

[b39] Moses D., Wang J., Yu G. & Heeger A. J. Temperature-independent photoconductivity in thin films of semiconducting polymers: Photocarrier sweep-out prior to deep trapping. Phys. Rev. Lett. 80, 2685–2688 (1998).

[b40] Auston D. H. Impulse-response of photoconductors in transmission-lines. IEEE J. Quantum Electron. 19, 639–648 (1983).

[b41] Liu Y. . Dependence of carrier mobility on nanocrystal size and ligand length in PbSe nanocrystal solids. Nano Lett. 10, 1960–1969 (2010). 20405957 10.1021/nl101284k

[b42] Choi J.-H. . Bandlike transport in strongly coupled and doped quantum dot solids: a route to high-performance thin-film electronics. Nano Lett. 12, 2631–2638 (2012). 22509936 10.1021/nl301104z

[b43] Lee J.-S., Kovalenko M. V., Huang J., Chung D. S. & Talapin D. V. Band-like transport, high electron mobility and high photoconductivity in all-inorganic nanocrystal arrays. Nat. Nano 6, 348–352 (2011). 10.1038/nnano.2011.46 21516091

[b44] Talapin D. V. & Murray C. B. PbSe nanocrystal solids for n- and p-channel thin film field-effect transistors. Science 310, 86–89 (2005). 16210533 10.1126/science.1116703

[b45] Klimov V. I. Optical nonlinearities and ultrafast carrier dynamics in semiconductor nanocrystals. J. Phys. Chem. B 104, 6112–6123 (2000).

[b46] Moreels I. . Composition and size-dependent extinction coefficient of colloidal PbSe quantum dots. Chem. Mater. 19, 6101–6106 (2007).

[b47] Schaller R. D. & Klimov V. I. Non-Poissonian exciton populations in semiconductor nanocrystals via carrier multiplication. Phys. Rev. Lett. 96, 097402 (2006). 16606314 10.1103/PhysRevLett.96.097402

[b48] McGuire J. A., Joo J., Pietryga J. M., Schaller R. D. & Klimov V. I. New aspects of carrier multiplication in semiconductor nanocrystals. Acc. Chem. Res. 41, 1810–1819 (2008). 19006342 10.1021/ar800112v

[b49] Maes W., De Meyer K. & Van Overstraeten R. Impact ionization in silicon: a review and update. Solid-State Electron 33, 705–718 (1990).

[b50] Zhitomirsky D. . Engineering colloidal quantum dot solids within and beyond the mobility-invariant regime. Nat. Commun. 5, 3803 (2014). 24801435 10.1038/ncomms4803

[b51] Bae W. K. . Highly effective surface passivation of PbSe quantum dots through reaction with molecular chlorine. J. Am. Chem. Soc. 134, 20160–20168 (2012). 23131125 10.1021/ja309783v

[b52] Gao J. B. . Quantum dot size dependent J-V characteristics in heterojunction ZnO/PbS quantum dot solar cells. Nano Lett. 11, 1002–1008 (2011). 21291196 10.1021/nl103814g

