# Supplementary material for: Carrier multiplication detected through transient photocurrent in device-grade films of lead selenide quantum dots
Source: Nat Commun. 2015 Sep 8;6:8185. doi: 10.1038/ncomms9185 (PMC4569798; doi:10.1038/ncomms9185)
Supplement: Supplementary Information — Supplementary Figures 1-3, Supplementary Notes 1-3 and Supplementary References. [file ncomms9185-s1.pdf]

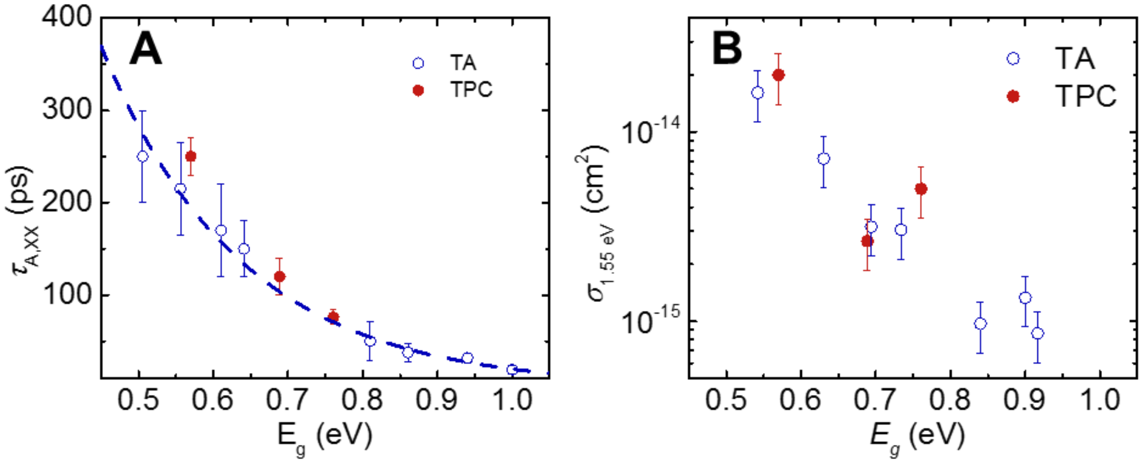

Supplementary Figure 1. (**A**) Biexciton Auger lifetimes as a function of band gap of PbSe QDs extracted from photocurrent transients (red) compared with literature values derived from transient absorption experiments (blue)[1]. Error bars are derived from the non-linear least squares fit to the data. (**B**) Absorption cross-sections derived from the fits of the measured pump-intensity-dependent fractions of single excitons (p_1) and biexcitons (p_2) (see example in Fig. 3B of the main text) to those for Poisson statistics of photon absorption events (red) compared to data from transient absorption experiments (blue). Error bars are standard deviations derived from the non-linear least squares fit to the data and the error in the measurement of the spot-size.

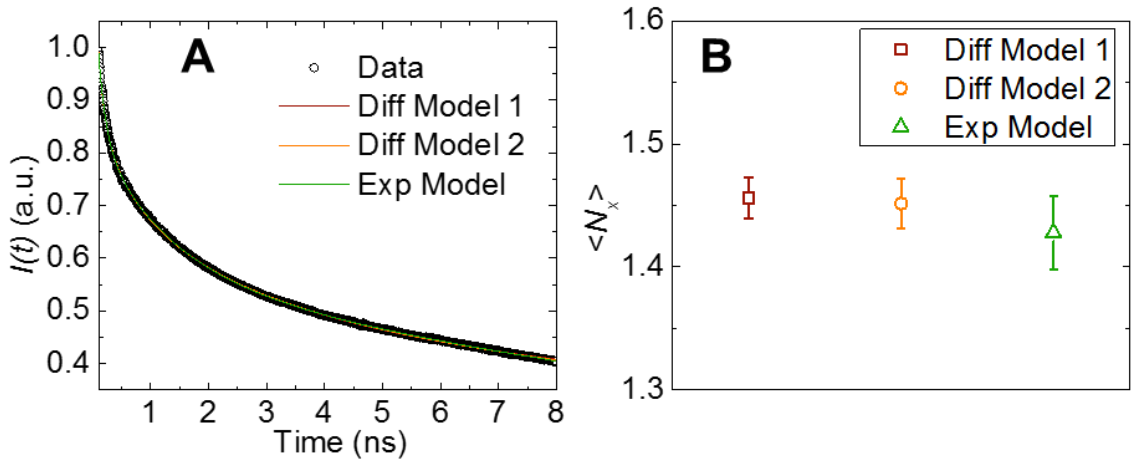

Supplementary Figure 2. Comparison of different models of the decay of the single exciton; see text for explanation of notations. (**A**) An experimental transient with fits to the three different models describing single exciton dynamics (see section "Modeling of photocurrent transients"). Experimental data correspond to an EDT treated film with a QD band gap of 0.69 eV excited at 1.55 eV with $<N_{abs}> = 0.6$ (**B**) Comparison of the exciton multiplicity $<N_X>$ derived from the fits in (**A**). The exciton multiplicity are indistinguishable within the error of the fits. Error bars are standard deviations derived from the non-linear least squares fit to the data.

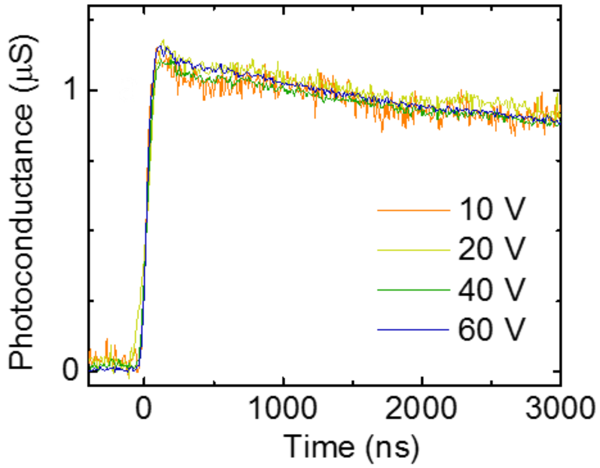

Supplementary Figure 3. TPC traces as a function of applied voltage (V) normalized by V; these measurements were conducted on the EDT treated film of PbSe QDs with E_g = 0.69 eV using 1.55 eV excitation with $\langle N_{abs} \rangle$ = 0.02. The fact the V-normalized traces are virtually indistinguishable indicates the photoconductance of the QD film is electric-field independent, that is, Ohmic.

Supplementary Notes

Supplementary Note 1: Modeling of photocurrent transients. Transient photocurrent (TPC) traces could be accurately modeled by the sum of two exponentials and an offset, convoluted with a Gaussian to account for the finite temporal resolution as is typical for experiments with slower temporal resolution[2]. Example fits can be seen in Fig. 3A in the main text. Prior to fitting the baseline signal at times before the pulse arrival, was subtracted. In some cases, the data was also filtered through a Fourier analysis to remove GHz noise arising from impedance mismatches in the device.

In typical optical experiments, the low fluence transients taken below the carrier multiplication (CM) threshold display a nearly constant signal for several nanoseconds. In this circumstance, the contribution of the multiexciton populations to the signal can be easily extracted by simply taking a ratio of the early time peak signal to the late-time "single-excitonic" signal measured at a few nanoseconds after completion of Auger decay[3]. However, in most of the measurements the recorded photocurrent transients exhibit a prominent nanosecond decay at low fluences which does not permit the usual data analysis strategy. Below, we briefly discuss different models which help elucidate the measured TPC dynamics. To explain the multiexponential decay, we consider a model case of a QD film containing two sub-ensembles of dots (fractions f_1 and f_2) with differing decay times of single excitons, arising from, *e.g.*, different numbers of surface defects. We further assume that both sub-ensembles have the same biexcitons lifetime. In this situation, we can describe the QD population dynamics with the following set of rate equations:

$$\frac{du_{XX1}}{dt} = -k_{A,XX} u_{XX1}, \ (1) \qquad \frac{du_{X1}}{dt} = k_{A,XX} u_{XX1} - k_1 u_{X1}, \ (2)$$

$$\frac{du_{XX2}}{dt} = -k_{A,XX} u_{XX2}, \ (3) \qquad \frac{du_{X2}}{dt} = k_{A,XX} u_{XX2} - k_2 u_{X2}. \ (4)$$

Here $u_{\mathrm{XX}i}(t)$ and $u_{\mathrm{X}i}(t)$ are the functions that describe temporal evolutions of biexction and single exciton populations of a sub-ensemble i (where $i = 1, 2$) with corresponding single exciton and biexciton decay rates k_i and $k_{\mathrm{A,XX}}$. The photocurrent density (j) then can be expressed as:

$$j(t) \propto f_1\left[2u_{\mathrm{XX1}}(t) + u_{\mathrm{X1}}(t)\right] + f_2\left[2u_{\mathrm{XX2}}(t) + u_{\mathrm{X2}}(t)\right]. \quad (5)$$

Here we take into consideration that biexcitons contribute twice the signal of a single exciton and further that the QD fractions in the two sub-ensembles are normalized to unity:

$$f_1 + f_2 = 1 \qquad (6)$$

Next we assume that the initial single-exciton and biexciton QD occupancies are identical for both sub-ensembles as defined by excitation fluence:

$$u_{\mathrm{XX1}}(0) = u_{\mathrm{XX2}}(0) = p_2, \qquad (7)$$

$$u_{\mathrm{X1}}(0) = u_{\mathrm{X2}}(0) = p_1. \qquad (8)$$

Equations (1)-(4) can be analytically solved and the resulting photocurrent can be presented as the sum of three exponential terms:

$$j(t)\ \mu\ \mathrm{A}\mathrm{e}^{-k_{\mathrm{A,XX}}t} + B\mathrm{e}^{-k_1 t} + C\mathrm{e}^{-k_2 t}. \quad (9)$$

In the limit that k_1 and k_2 are both much smaller than $k_{\mathrm{A,XX}}$ (as observed in our experiments), we find:

$$A = p_2, \qquad (10)$$

$$B = f_1(p_1 + p_2), \qquad (11)$$

$$C = f_2(p_1 + p_2). \qquad (12)$$

Using the latter expressions, we can re-write Eq. (9) as:

$$j(t)\ \mu\ p_2\mathrm{e}^{-k_{\mathrm{A,XX}}t} + \left(p_1 + p_2\right)\left(f_1\mathrm{e}^{-k_1 t} + f_2\mathrm{e}^{-k_2 t}\right), \quad (13)$$

or

$$j(t)\ \mu\ p_2\mathrm{e}^{-k_{\mathrm{A,XX}}t} + \left(p_1 + p_2\right)u_X, \qquad (14)$$

where $u_X = f_1\mathrm{e}^{-k_1 t} + f_2\mathrm{e}^{-k_2 t}$ is a functional form of the solution in the case when p_2 =0, that is, the photoexcited system is purely single-excitonic, the situation experimentally realized at low excitation fluences. From the experimental perspective, this implies that when there is a clear separation of biexciton and single-exciton timescales, a purely biexcitonic signal can be extracted from the measured high-pump-intensity time transients by subtracting a “tail-normalized” single-exciton trace measured at low fluences.

This subtractive procedure introduced in ref. 4, has been frequently used in transient absorption (TA) spectroscopy for isolating multiexciton dynamics. Another useful implication of this analysis is that the early time amplitudes of tail-normalized single-exciton and multiexciton traces can be used to directly evaluate the exciton multiplicity, $<N_X>$, and hence quantum efficiency of photon-to-exciton conversion when $<N_X>$ is measured in the limit of low fluences. Indeed, according to Eqs. (13) and (14), the $t = 0$ total signal amplitude is ($p_1 + 2p_2$), while the amplitude of the single-exciton component is ($p_1 + p_2$). The ratio of these amplitudes is $(p_1 + 2p_2)/(p_1 + p_2)$, which is the definition of $<N_X>$. Again, this procedure is similar to one used in CM studies utilizing ultrafast TA, where the exciton multiplicity is evaluated from the ratio of the early- to late-time TA signal[3].

To evaluate the range of validity of analytical solutions given by Eqs. (13) and (14) we have compared them to numerical solutions of Eqs. (1)-(4). We have found that for carrier relaxation parameters indicated by our measurements, the correction factor is less than 5%. However, when the k_i/k_A ratio becomes greater than 0.15 the correction can become significant.

We have additionally tested two other models, where we consider only one population and the single exciton rate equation was altered to one of the following forms:

$$\frac{du_X}{dt} = k_{A,XX}u_{XX} - \beta u_X^{\alpha} , \qquad (15),$$

$$\frac{du_X}{dt} = k_{A,XX}u_{XX} - \beta u_X^{\alpha} - k_1 u_X , \quad (16)$$

Where α is an arbitrary positive number. These rate equations were numerically solved and the resulting solutions were incorporate into Eq. (5) which then was used to fit TPC data to find initial QD occupancies. Shown in Supplementary Figure 2A we compare the fits of the three different models along with the resulting exciton multiplicity derived from the fits. Here, “diff model 1” refers to Eq. (15), “diff model 2” refers to Eq. (16), and “exp model” corresponds to Eqs. (1) to (4). On this timescale all three models accurately reproduce the data. The extracted exciton multiplicity derived from the fits in Supplementary Figure 2A are shown in Supplementary Figure 2B. We find that the extracted exciton multiplicity is virtually independent on the specific model used to describe the low fluence data. We conclude that the specific details of the decay of the photocurrent does not strongly influence our measurement, chiefly because there still remains a clear separation of timescales.

Supplementary Note 2: Poisson analysis of exciton multiplicities. To validate the derivation of probabilities p_1 and p_2 from the TPC traces, we have conducted a statistical analysis of a carrier distribution across a QD ensemble. When CM is inactive, it’s expected to follow Poisson statistics with average QD occupancy $<N> = <N_{abs}>$ and probabilities k_N of finding N excitons in a given QD expressed as $k_N = \frac{\langle N_{abs} \rangle^N}{N!} e^{-\langle N_{abs} \rangle}$ (*6*). With our limited time resolution we are not able to resolve Auger decay components due to triexcitons and multiexcitons of higher orders. However, as an ultimate product of decay of these species is a biexciton, they all contribute to probability p_2, which hence can be calculated from $p_2 = \sum_{i=2}^{\infty} k_i$. Probabilities p_0 and p_1 are simply equal to k_0 and k_1, respectively, which preserves the required normalization $p_0 + p_1 + p_2 = 1$. Using this truncated version of the Poisson distribution, we can

simultaneously fit pump-intensity dependence of p_1 and p_2 derived from the TPC traces using a QD absorption cross-section and a shared amplitude factor as two adjustable parameters (see Fig. 3B; main text). Using a truncated version of Poisson distribution we can also obtain the following expression for the exciton multiplicity:

$$\langle N_X \rangle = (2p_2 + p_1)/(p_1 + p_2) = 2 - \langle N_{abs} \rangle e^{-\langle N_{abs} \rangle}(1 - e^{-\langle N_{abs} \rangle})^{-1}. \quad (17)$$

This expression is used in the main text to model experimentally derived multiplicities in Fig. 3C, using the same absorption cross-section as in Fig 3B.

Supplementary Note 3: Estimating Carrier Multiplication Yield in Hydrazine Treated Films. For the EDT treated films we utilized the signatures of the Auger recombination of biexcitons to quantify the CM yield. However, for the EDT-Hydrazine treated films, low fluence data indicates the lack of Auger recombination which precludes this analysis. Alternatively, we may determine the CM yield from the peak value of the photocurrent through the use of equation (1) in the main text. Physically, the additional carrier generated through CM will increase the peak signal larger than expected from the input carrier density determined by the power in the optical pulse and absorbance of the film. Similar analysis strategies have been previously used in optical studies to quantify the CM yield[6]. Taking the ratio of equation (1) for two different wavelengths and evaluating at time zero we find:

$$\frac{j(h\nu_1)}{j(h\nu_2)} = \frac{q(h\nu_1)\,\mathrm{f}_{abs}(h\nu_1)}{q(h\nu_2)\,\mathrm{f}_{abs}(h\nu_2)}. \quad (18)$$

Here, we have assumed that the incident fluence is identical as in the experiments and $\mathrm{f}_{abs}(h\nu_1)$ is the fraction of light at frequency ν_1 absorbed. Utilizing the transients in Figure 4D, the absorbance spectra of the film, and assuming that the CM yield at 1.55 eV is zero, we find that the CM yield at 3.1 eV is 148 % for the hydrazine-treated films**,** which is in excellent agreement with the yield measured for the EDT-treated film (144%).

Supplementary References

1 McGuire, J. A., Joo, J., Pietryga, J. M., Schaller, R. D. & Klimov, V. I. New Aspects of Carrier Multiplication in Semiconductor Nanocrystals. *Acc. Chem. Res.* **41**, 1810-1819 (2008).

2 Sandberg, R. L. *et al.* Multiexciton Dynamics in Infrared-Emitting Colloidal Nanostructures Probed by a Superconducting Nanowire Single-Photon Detector. *ACS Nano* **6**, 9532-9540 (2012).

3 Schaller, R. D. & Klimov, V. I. High efficiency carrier multiplication in PbSe nanocrystals: Implications for solar energy conversion. *Phys. Rev. Lett.* **92**, 186601 (2004).

4 Klimov, V. I., Mikhailovsky, A. A., McBranch, D. W., Leatherdale, C. A. & Bawendi, M. G. Quantization of Multiparticle Auger Rates in Semiconductor Quantum Dots. *Science* **287**, 1011-1013 (2000).

5 Klimov, V. I. Optical nonlinearities and ultrafast carrier dynamics in semiconductor nanocrystals. *J. Phys. Chem. B* **104**, 6112-6123 (2000).

6. Trinh, M. T. *et al*. Direct generation of multiple excitons in adjacent silicon nanocrystals revealed by induced absorption. *Nat Photonics* **6**, 316-321 (2012).
